# Tumor Necrosis Factor (*TNF*) –308G>A, Nitric Oxide Synthase 3 (*NOS3*) +894G>T Polymorphisms and Migraine Risk: A Meta-Analysis

**DOI:** 10.1371/journal.pone.0129372

**Published:** 2015-06-22

**Authors:** Min Chen, Wenjing Tang, Lei Hou, Ruozhuo Liu, Zhao Dong, Xun Han, Xiaofei Zhang, Dongjun Wan, Shengyuan Yu

**Affiliations:** 1 Department of Neurology, Chinese PLA General Hospital, Beijing, China; 2 Department of Neurology, the First People’s Hospital of Nanyang, Nanyang, Henan Province, China; FIOCRUZ, BRAZIL

## Abstract

**Background and Objective:**

Conflicting data have been reported on the association between tumor necrosis factor (*TNF*) –308G>A and nitric oxide synthase 3 (*NOS3*) +894G>T polymorphisms and migraine. We performed a meta-analysis of case-control studies to evaluate whether the *TNF* –308G>A and *NOS3 *+894G>T polymorphisms confer genetic susceptibility to migraine.

**Method:**

We performed an updated meta-analysis for *TNF* –308G>A and a meta-analysis for *NOS3 *+894G>T based on studies published up to July 2014. We calculated study specific odds ratios (OR) and 95% confidence intervals (95% CI) assuming allele contrast, dominant model, recessive model, and co-dominant model as pooled effect estimates.

**Results:**

Eleven studies in 6682 migraineurs and 22591 controls for *TNF* –308G>A and six studies in 1055 migraineurs and 877 controls for *NOS3* +894G>T were included in the analysis. Neither indicated overall associations between gene polymorphisms and migraine risk. Subgroup analyses suggested that the “A” allele of the *TNF* –308G>A variant increases the risk of migraine among non-Caucasians (dominant model: pooled OR = 1.82; 95% CI 1.15 – 2.87). The risk of migraine with aura (MA) was increased among both Caucasians and non-Caucasians. Subgroup analyses suggested that the “T” allele of the *NOS3* +894G>T variant increases the risk of migraine among non-Caucasians (co-dominant model: pooled OR = 2.10; 95% CI 1.14 – 3.88).

**Conclusions:**

Our findings appear to support the hypothesis that the *TNF *–308G>A polymorphism may act as a genetic susceptibility factor for migraine among non-Caucasians and that the *NOS3* +894G>T polymorphism may modulate the risk of migraine among non-Caucasians.

## Introduction

Migraine is characterized by recurrent, moderate to severe, throbbing headache attacks, associated with photophobia, phonophobia, nausea, and vomiting. Migraine is remarkably common, affecting approximately 11% of the adult population around the world [1] and 9.3% in China [2]. This condition is associated with high financial costs, reported to cost $18.5 billion Euros per year in Europe [3], and is listed as one of the top 20 most debilitating disorders according to the World Health Organization [4]. Migraine is therefore a public health problem that has a major impact on both the individual and society.

The etiology of migraine is complex, involving both multiple genetic and environmental factors [5]. It is now acknowledged that changes in immune homeostasis leading to changes in cytokine profiles can contribute to migraine [6].

Sterile meningeal inflammation is a key mechanism that may underlie the sustained activation and sensitization of perivascular meningeal nociceptors. Cytokines and nitric oxide (NO) have been implicated in the pathogenesis of migraine in both animal and human studies [7].

Tumor necrosis factor-alpha (TNF-α) is an important cytokine that can promote powerful hyperalgesia by causing prostanoid release, increasing nerve growth factor (NGF) and bradykinin receptor expression, or by modulation of activity within sympathetic fibers. Several studies have indicated changes in serum [8], plasma [9], and urine [10] concentrations of TNF-α as well as altered serum concentrations of the soluble TNF-α receptor [11] among migraineurs either during or outside of attacks. Furthermore, TNF-α can stimulate transcription of calcitonin gene-related peptide (CGRP), which plays a pivotal role in the pathophysiology of migraine [12]. Exaggerated serum concentrations of TNF reverted to normality after drug therapy [13].

NO is a potent endogenous vasodilator, which is a key molecule affecting the pain associated with migraine [14], as it was shown to cause immediate headache in migraineurs and less often in control subjects [15–17]. Impaired release of NO can lead to vascular/coagulative dysfunction [18] with subsequent variation in cerebral blood flow, and activate cortical spreading depression and the trigeminovascular system, which mediate the pain [19].

NO is synthesized from l-arginine and molecular oxygen by the NO synthase (NOS) family. In endothelial cells, NO is synthesized by the endothelial NOS (eNOS). Deregulation of eNOS influences the plasma level of NO, which has an anti-inflammatory effect. The haplotype of the *NOS3* gene is associated with variability in endogenous NO formation [20].

Migraine is a complex genetic disorder in which several genes play a role [21–23]. Identification of susceptibility genes will enable a better understanding of the mechanisms underlying the disease processes. The gene encoding TNF-α is located in the class III gene cluster of the major histocompatibility complex (MHC) on chromosome 6p21.3. eNOS is encoded by a gene of 26 exons (*NOS3*) located on chromosome 7 [17]. Variants in these genes have been shown to modulate production of TNF-α [24] and NO [20, 25]. Therefore, various polymorphisms in *TNF* and *NOS3* among migraineurs have been investigated. The most widely studied variants are *TNF* –308G>A and *NOS3* +894G>T. A number of recent observations concerning the association between *TNF* –308G>A and *NOS3* +894G>T and migraine support a significant genetic component to predisposition toward this frequently undiagnosed disabling disorder [26–33], but the results are controversial [34–38]. A single study may not have sufficient power to completely demonstrate this complicated genetic relationship because of relatively small sample sizes, and larger studies could overcome these disadvantages. There have been two previous meta-analyses of *TNF* –308G>A, and the scenario remained virtually unchanged [39, 40]. To clarify this association, we performed a meta-analysis of case-control studies to evaluate whether the *TNF* –308G>A and *NOS3* +894G>T polymorphisms confer genetic susceptibility to migraine.

## Materials and Methods

### Literature sources and search strategy

**Fig 1:**
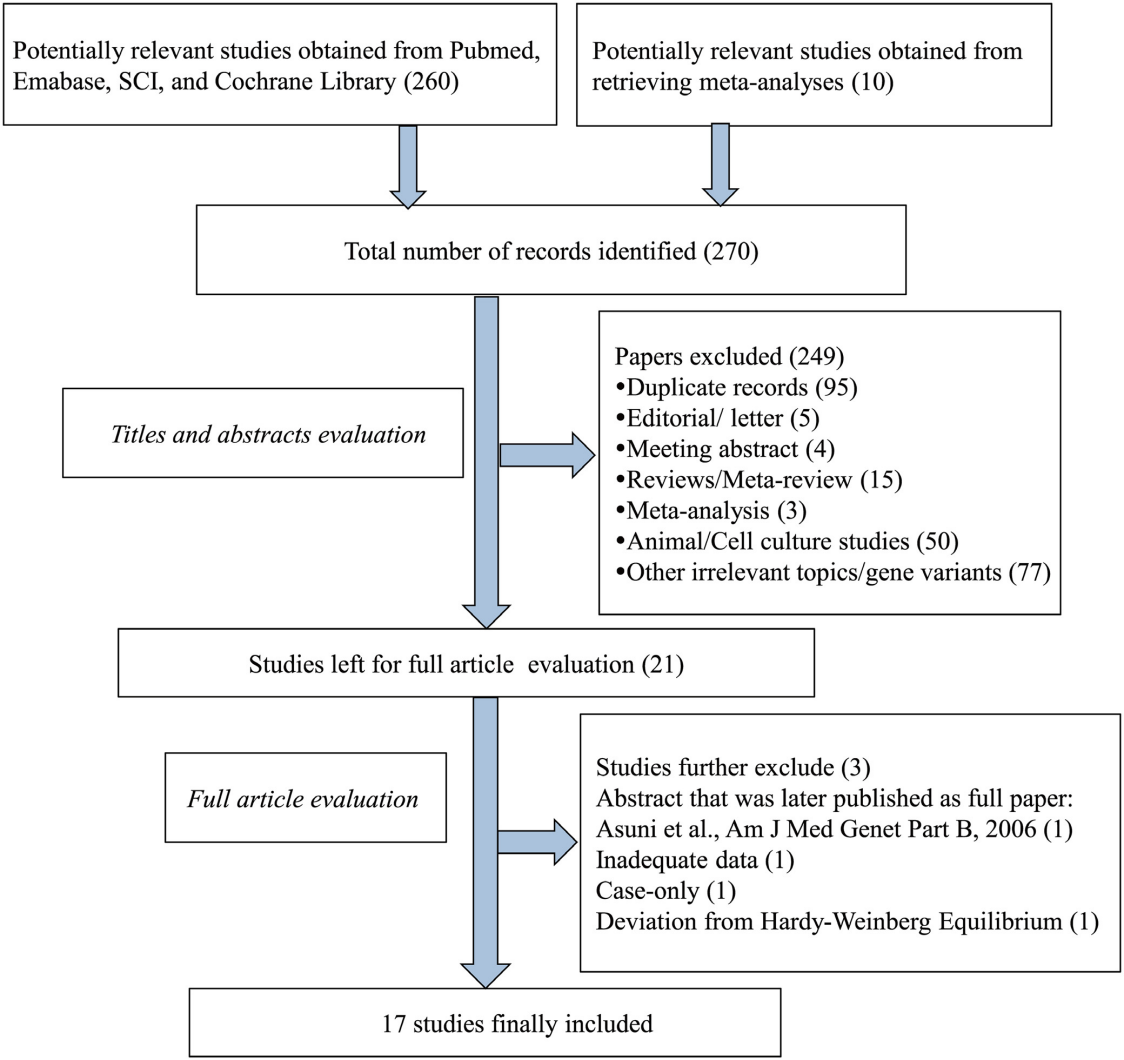
Flow chart of the study selection process.

Two of the authors (MC and WT) searched the PubMed, EMBASE, Science Citation Index (SCI), and Cochrane Library electronic databases for tumor necrosis factor and endothelial nitric oxide synthase (“tumor necrosis factor-α” OR ‘‘tumor necrosis factor-alpha” OR “TNF-α” OR “TNF-alpha” OR “endothelial nitric oxide synthase” OR “eNOS”) AND “headache” OR “headache disorder” OR “migraine” OR “migraine disorder” OR “migraine with aura” OR “migraine without aura” AND “gene” OR “polymorphism” OR “genetic variation” OR “polymorphisms” OR “rs1800629” OR *TNF* –308G>A” OR “rs1799983” OR “Glu298Asp” OR “*NOS3* +894G/T.” The search was performed without any restrictions, except that the studies were conducted in humans. The full electronic search strategy of *TNF* –308G>A for PubMed was (“migraine disorders” [MeSH Terms] OR “migraine disorders” [All Fields] OR “migraine” [All Fields] OR “migraine disorder” [All Fields] OR “headache disorders” [MeSH Terms] OR “headache disorders” [All Fields] OR “headache” [All Fields] OR “headache disorder” [All Fields])) OR “migraine with aura” [MeSH Terms] OR “migraine with aura” [All Fields] OR “migraine without aura” [MeSH Terms] OR “migraine without aura” [All Fields]) AND (“tumor necrosis factor-alpha” [MeSH Terms] OR “tumor necrosis factor-alpha” [All Fields] OR “tumor necrosis factor alpha” [All Fields] OR “tumor necrosis factors” [MeSH Terms] OR “tumor necrosis factors” [All Fields] OR “tnf” [All Fields] OR “tnf alpha” [All Fields]) AND (“genetic” [All Fields] OR “genetics” [All Fields] OR “genetics” [MeSH Terms] OR “polymorphism,” “genetic” [MeSH Terms] OR “genetic polymorphism” [All Fields] OR (“polymorphism” [All Fields] AND “genetic” [All Fields]) OR “variant” [All Fields] OR “variants” [All Fields] OR “rs1800629” OR “*TNF* –308G>A”) AND “humans” [MeSH Terms]. In addition, the reference lists of selected papers and potentially relevant studies in the previous meta-analyses were also screened to further identify potentially relevant papers through reference association. The last search was updated in July 2014. The selection process is shown as a flow chart in Fig 1.

### Inclusion criteria

The meta-analysis included only articles (i) that evaluated the association of the *TNF* –308G>A or *NOS3* +894G>T polymorphism and migraine risk based on a case-control or cohort design, and (ii) that contained information on the sample sizes, distribution of genotypes, and allele frequencies for the polymorphisms investigated among migraineurs and non-migraineurs allowing for calculation of crude risks for migraine. In publications with overlapping cases and/or controls, the most recent or largest population was chosen.

In the first step of the analysis, two of the investigators (MC and WT) independently identified all studies not meeting any of the pre-specified criteria by screening the titles and abstracts, and these studies were excluded. In the second step, the same investigators independently evaluated the full-text publications of the remaining studies. Studies were excluded if they did not meet all of the criteria. Any discrepancies were adjudicated by a third reviewer (ZD).

### Exclusion criteria

Studies were excluded if one of the following existed: (1) studies with insufficient genotyping data of the participants; (2) case-only studies; (3) review articles; (4) animal studies; (5) editorials; (6) abstracts; (7) studies that reported associations between gene polymorphisms and recovery from a migraine attack; (8) studies that reported associations between gene polymorphisms and response to any therapy; (9) studies that deviated from Hardy—Weinberg equilibrium.

### Data extraction and contact with authors

Two of the investigators (MC and WT) independently reviewed, extracted, and entered data from the studies in a standardized data extraction form. Discrepancies were resolved by consensus. The items recorded were authors’ names, year of publication, ethnicity of subjects, study design, genotyping method, migraine status (migraine, migraine with aura [MA], and migraine without aura [MO]), diagnostic criterion of cases, gender of individuals in the study population, study size, allele and genotype frequencies, and additional potentially relevant information. We attempted to collect genetic information for all migraineurs and migraineurs with and without aura separately as well as for the whole study population and for females and males separately. If not presented in the article, allele and genotype frequencies were calculated when possible. For studies that did not allow extraction of all relevant information, including genotype and allele frequencies, we e-mailed the corresponding authors to obtain the missing information. Authors not responding within 1 week were sent up to two reminders by e-mail.

### Quality score assessment

The quality of studies was assessed by the same two reviewers (MC and WT) independently using a modified 10-point Newcastle—Ottawa Scale (NOS) (http://www.ohri.ca/programs/clinical_epidemiology/oxford.asp). The quality of each study was assessed and awarded stars based on indicators of quality, including selection of study population, comparability, and exposure assessment independently. The quality score ranged from 0 (worst) to 10 points (best). Any discrepancies were adjudicated by a third reviewer (LH).

### Statistical methods

All analyses were performed using STATA version 12.0 (Stata Corporation, College Station, TX). Unless otherwise stated, P < 0.05 was taken to indicate statistical significance. Hardy—Weinberg equilibrium (HWE) for each study was determined using the goodness-of-fit *χ* ^2^ test. The strengths of the associations between the *TNF* –308G>A and *NOS3* +894G>T polymorphisms and migraine risk were examined by ORs and corresponding 95% CIs. The pooled ORs were calculated for co-dominant model (AA vs. GG and GA vs. GG for *TNF* –308G>A, TT vs. GG and GT vs. GG for *NOS3* +894G>T), dominant model (AA+GA vs. GG for *TNF* –308G>A, TT+GT vs. GG for *NOS3* +894G>T), recessive model (AA vs. GA + GG for *TNF* –308G>A, TT vs. GT+GG for *NOS3* +894G>T), and allele contrast (A vs. G for *TNF* –308G>A, T vs. G for *NOS3* +894G>T). Heterogeneity across studies was detected by the *χ* ^2^ test-based Q-statistic (P < 0.10 was deemed as evidence of heterogeneity) as well as *I* ^*2*^ statistic, which takes values between 0% and 100% (*I* ^*2*^ < 25% represents absence of heterogeneity; *I* ^*2*^ = 25%– 50% moderate heterogeneity; *I* ^*2*^ > 50% large heterogeneity) [41]. When the heterogeneity was indicated to be non-significant (P > 0.10), the fixed-effects model (Mantel—Haenszel method) [42] was applied; otherwise the random-effects model (DerSimonian and Laird method) [43] was performed. Stratified analyses were performed by ethnicity, type of migraine, and gender to handle heterogeneity. We used Galbraith plots to visually examine the impact of individual studies on the overall homogeneity test statistic. Leave-one-out sensitivity analysis was carried out to determine the extent of influence of single studies on the combined results. Begg’s funnel test and Egger’s test were applied to evaluate publication bias across studies (P < 0.10 was taken to indicate statistical significance).

## Results

### Eligible studies

Fig 1. summarizes the process of identifying eligible studies. A total of 270 potential studies were identified by electronic and manual searches. After reviewing titles and abstracts, 249 were removed. Among the remaining 21 papers, 12 papers focused only on *TNF* –308G>A [26, 28–31, 34–37, 44–46], eight focused only on *NOS3* +894G>T [33, 38, 47–52], and one dealt with both *TNF* –308G>A and *NOS3* +894G>T [27]. After further examination, among the 12 papers investigating only *TNF* –308G>A, we excluded one abstract [46] because detailed results were later published in full [37]. Another study was excluded because of deviation from HWE and the method used for SNP genotyping (SSP-PCR), which is particularly susceptible to errors [29]. Among the eight papers regarding only *NOS3* +894G>T, one was excluded because no information about the distribution of genotypes was provided [47], and one was excluded because of its case-only design and therapy response evaluation [52]. In the paper regarding both *TNF* –308G>A and *NOS3* +894G>T [27], information about *TNF* –308G>A was available but not that for *NOS3* +894G>T. MC and WT independently read the articles, and 11 studies were finally included for *TNF* –308G>A and 6 studies were included for *NOS3* +894G>T. Kappa statistic for agreement of *TNF* –308G>A between the two reviewers was 0.9, and that for *NOS3* +894G>T was 0.86. Any discrepancies were adjudicated by a third reviewer (ZD).

### Contact with authors

Among the 11 papers focusing on *TNF* –308G>A, 7 [27, 28, 31, 34, 36, 37, 44] reported genotype and allele frequencies for all participants and for overall migraine, as well as stratified by gender and aura; however, the remaining 4 papers did not [26, 30, 35, 45]. After contacting the authors of these papers, we obtained complete data for one study [35]. Among the eight papers discussing *NOS3* +894G>T, six reported genotype and allele frequencies for all participants and for overall migraine as well as those stratified by aura [33, 38, 49–51, 53], while the remaining two did not [27, 47]. We attempted to contact the authors, but they did not respond and so we excluded these two studies from the analysis.

### Study characteristics

The detailed characteristics of each study included in the meta-analysis are presented in Table 1. These studies were published between 2002 and 2014. In one study, migraine was self-reported [27], while the rest were based on recognized diagnostic criteria [26, 28–31, 33–36, 38, 44, 45, 48–51, 54]. Among the studies included in the analysis, 11 evaluated *TNF* –308G>A polymorphisms [26–31, 34–37, 44, 45] and 6 evaluated *NOS3* +894G>T polymorphisms [33, 50, 51, 53, 55, 56].

**Table 1:** Characteristics of the eligible studies in this meta-analysis.

First Author (year)	Ethnicity	Gender	Sample size of controls	Sample size of cases	Diagnostic criteria of cases	Design of study	Detecting method of polymorphism	Source of controls	HWE	mNOS (*)
(A) Studies of *TNF* –308G>A polymorphism										
Trabace, 2002 [32]	Caucasians	F/M	101	79	IHS	Case-control	PCR-RFLP	Population	Y	7
Rainero, 2004 [26]	Caucasians	F/M	306	299	IHS	Case-control	PCR-RFLP	Blood donors	Y	7
Herken, 2005 [45]	Non-Caucasians	F/M	60	60	IHS	Case-control	PCR-RFLP	Population	Y	6
Lee, 2007 [34]	Non-Caucasians	F	382	439	IHS	Case-control	PCR	Not mentioned	Y	6
Asuni, 2009 [37]	Caucasians	F/M	278	299	IHCD-II	Case-control	PCR-RFLP	Blood donors	Y	7
Schurks, 2009 [65]	Caucasians	F	20,425	4577	Self-reported	Cohort	PCR	Population	Y	6
Ghosh, 2010 [31]	Non-Caucasians	F/M	216	216	IHS	Case-control	PCR-RFLP	Population	Y	7
Yilmaz, 2010 [28]	Non-Caucasians	F/M	96	67	IHCD-II	Case-control	PCR-RFLP	Population, hospital,family members of patients	Y	6
Pappa, 2010 [36]	Caucasians	F/M	178	103	IHCD-II	Case-control	PCR-RFLP	Population	Y	8
Ates, 2011 [30]	Non-Caucasians	F/M	202	203	IHS	Case-control	ARMS-PCR	Hospital	Y	6
Stuart, 2013[35]	Caucasians	F/M	345	340	IHS	case-control	SNPs	Hospital	Y	6
(B) Studies for *NOS3* +894G>T polymorphism										
Borroni, 2006 [33]	Caucasians	F/M	125	156	IHS	Case-control	PCR	Healthy volunteers	Y	6
Toriello, 2008 [38]	Caucasians	F/M	337	341	IHS	Case-control	R-T PCR	Patients’ friends and healthy volunteers.	Y	6
Gruber, 2010 [53]	Caucasians	F/M	76	54	IHS	Case-control	R-T PCR	Patients’ friends and healthy volunteers	Y	6
Goncalves, 2011 [49]	Non-Caucasians	F	117	178	IHS	Case-control	RT-PCR	Population	Y	7
Goncalves, 2012 [50]	Non-Caucasians	F	99	150	IHS	Case-control	RT-PCR	Population	Y	7
Eroz, 2014 [51]	Non-Caucasians	F/M	123	176	IHCD-II	Case-control	PCR-RFLP	Blood donors	Y	4

ICHD-II: International Classification of Headache Disorders-II; HIS: International Headache Society; HWE: Hardy-Weinberg equilibrium; PCR: polymerase chain reaction; RFLP: restriction fragment length polymorphism; ARMS: amplification refractory mutation system; SNPs: single nucleotide polymorphisms; RT-PCR: real-time polymerase chain reaction.

Y: consistent with HWE; F: female; M: male. NOS: Newcastle—Ottawa Quality Assessment Scale for Case Control Studies.

For *TNF* –308G>A, all studies were of a case-control design [26, 28–31, 34–36, 44–46] except one that had a cohort design [27]. One study was performed in children [36], and the others in adults [26–32, 34, 35, 45, 46]. Six studies were performed in Caucasians [26, 27, 32, 35, 36, 46] and five in non-Caucasians [28, 30, 31, 34, 45]. For *NOS3* +894G>T, all studies were of a case-control design, and all studies were performed in adults. Three studies were performed in Caucasians [33, 53, 56] and three in non-Caucasians [50, 51, 55].

The distributions of genotypes and alleles for *TNF* –308G>A and *NOS3* +894G>T among migraineurs and controls are listed in S1 and S2 Tables.

Tables 2 and 3 summarize the pooled effect estimates, measures of heterogeneity, and tests for *TNF* –308G>A and *NOS3* +894G>T polymorphisms, respectively.

**Table 2:** Main results of the pooled data for the *TNF* –308G>A polymorphism.

Subgroups		AA vs. GG			AA+GA vs. GG			AA vs. GA+GG			A vs. G			GA vs. GG		
		OR (95% CI)	P_h_	*I* ^*2*^ (%)	OR (95% CI)	P_h_	*I* ^*2*^ (%)	OR (95% CI)	P_h_	*I* ^*2*^ (%)	OR (95% CI)	P_h_	*I* ^*2*^ (%)	OR (95% CI)	P_h_	*I* ^*2*^ (%)
Total		0.84 (0.45,1.56)	0.080	41.7	1.14 (0.84,1.55)	0.000	83.2	0.92 (0.77,1.11)	0.143	33.1	1.11 (0.83,1.47)	0.000	84.8	1.15 (0.86,1.55)	0.000	80.1
Ethnicity																
Caucasians		0.91 (0.76,1.09)	0.204	30.9	0.81 (0.56,1.17)	0.015	67.8	0.90 (0.75,1.08)	0.280	20.3	0.79 (0.56,1.12)	0.000	84.3	0.85 (0.59, 1.20)	0.037	60.8
Non-Caucasians		1.60 (0.20,12.5)	0.063	58.8	**1.82 (1.15,2.87)**	0.000	84.3	1.46 (0.21,10.2)	0.088	54.1	**1.74 (1.13,2.67)**	0.012	69.1	**1.78 (1.17,2.72)**	0.000	81.8
Type of migraine																
MA	Total	1.16 (0.87,1.54)	0.960	0	**1.17 (1.05,1.30)**	0.195	30.6	1.11 (0.84,1.48)	0.956	0	**1.13 (1.03,1.24)**	0.299	17.1	**1.17 (1.05,1.31)**	0.123	40.2
	Caucasians	1.16 (0.87,1.55)	0.806	0	**1.15 (1.03,1.28)**	0.335	11.7	1.12 (0.84,1.49)	0.799	0	**1.12 (1.02,1.23)**	0.403	0	**1.15 (1.02,1.29)**	0.256	25.9
	Non-Caucasians	0.93 (0.11,8.20)	0.967	0	**1.62 (1.03,2.53)**	0.230	31.9	0.84 (0.10,7.42)	0.990	0	1.49 (0.97,2.28)	0.284	20.5	**1.69 (1.08,2.65)**	0.212	35.4
MO	Total	0.89 (0.43,1.82)	0.088	40.4	0.98 (0.69,1.38)	0.000	80.4	0.96 (0.75,1.22)	0.156	31.6	0.98 (0.70,1.36)	0.000	82.4	0.99 (0.71,1.37)	0.000	76.1
	Caucasians	0.88 (0.68,1.14)	0.275	21.1	0.74 (0.49,1.12)	0.000	81.5	0.90 (0.69,1.16)	0.367	7.7	0.73 (0.49,1.09)	0.000	83.1	0.77 (0.52,1.15)	0.000	77.6
	Non-Caucasians	2.41 (0.34,17.1)	0.086	54.5	1.65 (0.922.97)	0.043	63.1	2.48 (0.91,6.76)	0.110	50.2	1.65 (0.92,2.99)	0.019	69.8	**1.53 (1.11,2.11)**	0.116	49.3
Gender																
female	Migraine	0.95 (0.79,1.15)	0.133	35.7	1.13 (0.80,1.58)	0.000	79.8	0.94 (0.78,1.1)	0.210	26.3	1.09 (0.80, 1.50)	0.000	81.5	1.14 (0.82,1.57)	0.000	75.8
	MA	1.16 (0.85,1.60)	0.981	0	**1.65 (1.02,2.68)**	0.067	58.1	1.11 (0.81,1.5)	0.965	0	1.16 (1.05,1.28)	0.146	44.2	**1.80 (1.03,3.14)**	0.032	65.9
	MO	1.02 (0.78,1.33)	0.319	14.5	1.23 (0.87,1.74)	0.018	60.8	1.02 (0.78,1.3)	0.435	0	1.21 (0.85,1.72)	0.006	66.5	1.21 (0.88,1.65)	0.061	50.1
male	Migraine	0.76 (0.32,1.81)	0.685	0	0.83 (0.58,1.18)	0.179	32.7	0.76 (0.32,1.8)	0.786	0	0.81 (0.59,1.12)	0.122	40.3	0.87 (0.60,1.26)	0.302	16.8
	MA	*	*	*	1.06 (0.28,4.09)	0.623	0	*	*	*	1.02 (0.28,3.74)	0.710	0	1.10 (0.28,4.24)	0.552	0
	MO	1.61 (0.35,7.35)	0.872	0	0.97 (0.55,1.70)	0.456	0	1.61 (0.35,7.3)	0.912	0	0.96 (0.57,1.63)	0.352	9.5	0.99 (0.56,1.75)	0.588	0

MA: migraine with aura; MO: migraine without aura; OR: odds ratio; 95% CI: 95% confidence interval;

*effect estimates for some studies could not be calculated due to the small number of studies.

**Table 3:** Main results of the pooled data for the *NOS3* +894G>T polymorphism.

Subgroups		TT vs. GG			TT+GT vs. GG			TT vs. GT+GG			T vs. G			GT vs. GG		
		OR (95% CI)	P_h_	*I* ^*2*^ (%)	OR (95% CI)	P_h_	*I* ^*2*^ (%)	OR (95% CI)	P_h_	*I* ^*2*^ (%)	OR (95% CI)	P_h_	*I* ^*2*^ (%)	OR (95% CI)	P_h_	*I* ^*2*^ (%)
Total		1.29 (0.95,1.75)	0.372	6.9	1.03 (0.66,1.60)	0.000	80.8	1.27 (0.96,1.68)	0.639	0	1.05 (0.82,1.34)	0.015	64.4	0.97 (0.59,1.59)	0.000	82.8
Ethnicity																
Caucasians		1.08 (0.76,1.54)	0.699	0	0.96 (0.75,1.24)	0.000	0	1.13 (0.82,1.56)	0.514	0	1.02 (0.86,1.21)	0.827	0	0.93 (0.71,1.21)	0.625	0
Non-Caucasians		**2.10 (1.14,3.88)**	0.534	0	1.09 (0.40,2.99)	0.002	92.1	**1.84 (1.02,3.33)**	0.953	0	1.11 (0.63,1.94)	0.002	84.5	0.99 (0.33,2.99)	0.000	92.9
Type of migraine																
MA	Total	**1.61 (1.12,2.31)**	0.700	0	1.10 (0.65,1.87)	0.001	76.4	**1.50 (1.08,2.09)**	0.782	0	1.18 (0.90,1.55)	0.057	53.4	0.97 (0.52,1.79)	0.000	79.8
	Caucasians	1.43 (0.94,2.17)	0.665	0	1.11 (0.81,1.51)	0.996	0	1.41, (0.98,2.04)	0.450	0	1.17 (0.95,1.44)	0.787	0	0.99 (0.71,1.39)	0.775	0
	Non-Caucasians	**2.37 (1.12,4.99)**	0.656	0	1.07 (0.30,3.88)	0.000	90.5	1.93 (0.94 3.97)	0.866	0	1.11 (0.56,2.19)	0.007	80.0	0.93 (0.21,4.02)	0.000	91.6
MO	Total	1.06 (0.73,1.54)	0.233	26.9	0.94 (0.64,1.39)	0.012	65.7	1.10 (0.78, 1.55)	0.390	4.1	0.97 (0.75,1.24)	0.058	53.1	0.93 (0.61,1.42)	0.008	68.3
	Caucasians	0.80 (0.51,1.27)	0.408	0	0.84 (0.62,1.14)	0.832	0	0.89 (0.59, 1.35)	0.299	17.1	0.89 (0.71,1.10)	0.624	0	0.86 (0.62,1.19)	0.627	0
	Non-Caucasians	1.90 (0.97,3.73)	0.625	0	1.05 (0.46,2.394)	0.001	85.3	1.81 (0.95, 3.47)	0.973	0	1.09 (0.67,1.78)	0.018	75.2	0.96 (0.39,2.35)	0.001	86.4

MA: migraine with aura; MO: migraine without aura; OR: odds ratio; 95% CI: 95% confidence interval

### Association between *TNF* –308G>A polymorphism and migraine

**Fig 2:**
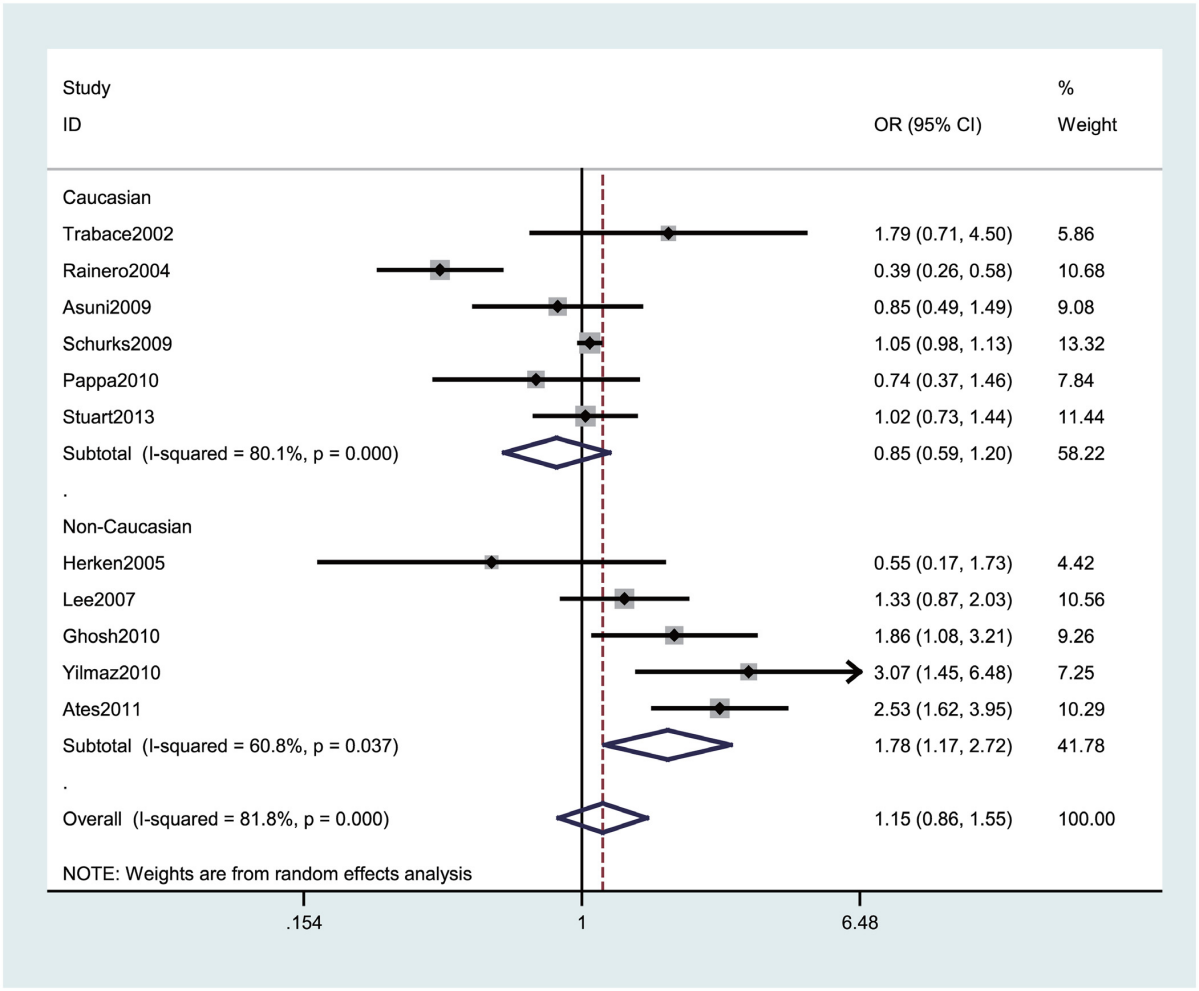
Forest plot of migraine risk associated with the *TNF* –308G>A polymorphism stratified by ethnicity under the GA vs. GG model. The boxes and horizontal lines represent the OR and the corresponding 95% CI. The areas of the boxes indicates the weight (inverse of the variance). The diamonds correspond to the summary OR and 95% CI.

**Fig 3:**
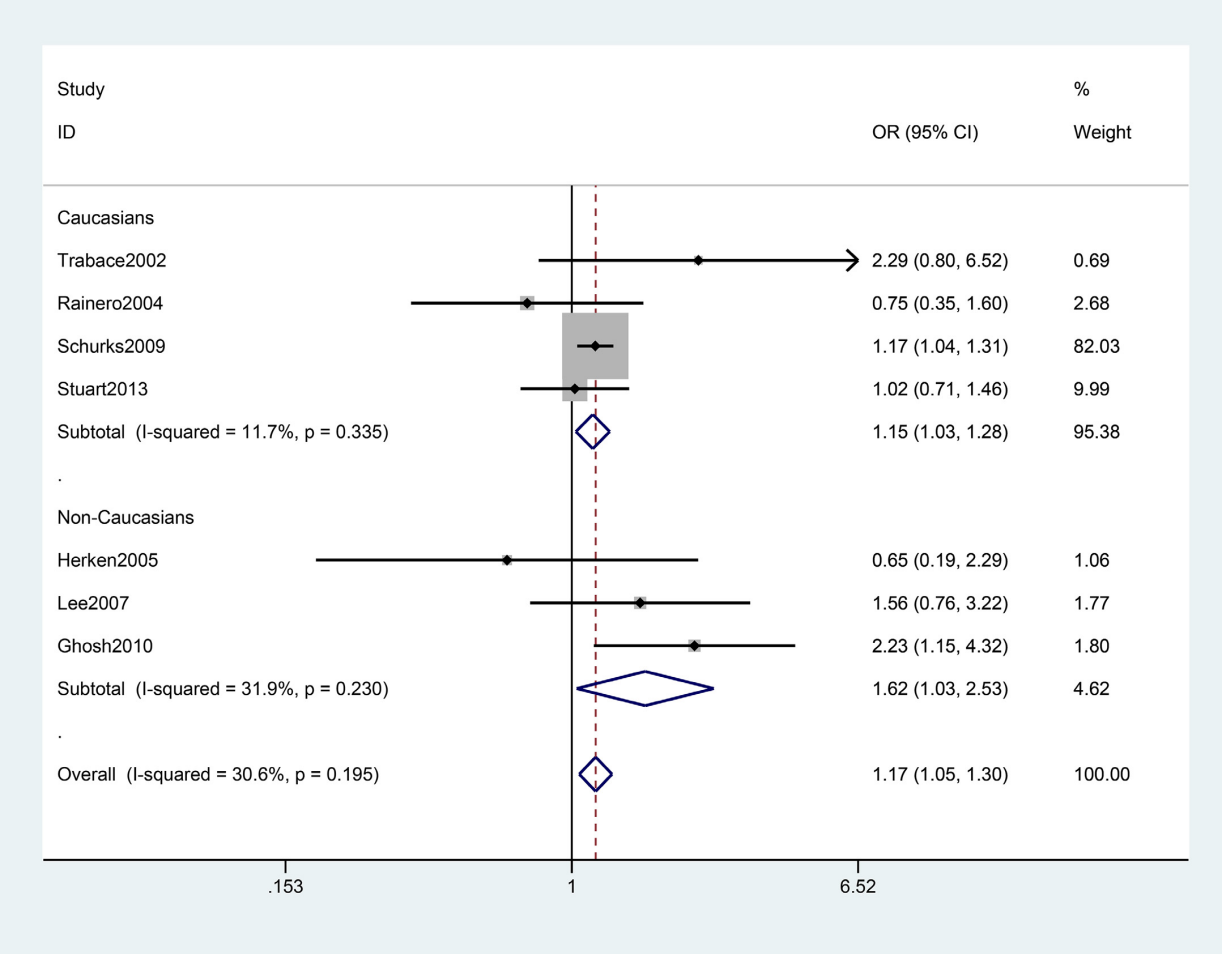
Forest plot of migraine with aura risk associated with the *TNF* –308G>A polymorphism stratified by ethnicity under the AA+GA vs. GG model. The boxes and horizontal lines represent the OR and the corresponding 95% CI. The areas of the boxes indicate the weight (inverse of the variance). The diamonds correspond to the summary OR and 95% CI.

Table 2 lists the main meta-analysis results, which were calculated with the fixed-effects model when Ph > 0.10 and the random-effects model when the Ph < 0.10. Based on data from 11 studies for *TNF* –308G>A with 6682 genotyped migraine cases and 22591 controls, no significant association between *TNF* –308G>A polymorphism and migraine risk was observed under any of the genetic models. Strikingly, the meta-analysis provided an OR of 1.74 (95% CI: 1.13–2.67, Ph = 0.012) under A vs. G, an OR of 1.82 (95%CI: 1.15–2.87, Ph = 0.000) under AA+GA vs. GG, and an OR of 1.78 (95% CI: 1.17–2.72, Ph = 0.000) under GA vs. GG among non-Caucasians, while none of the contrast models showed a significant association in Caucasian populations (Fig 2). When stratifying the data by type of migraine, we observed an increased risk of migraine with aura (MA) among all participants (AA+GA vs. GG: pooled OR = 1.17, 95% CI: 1.05–1.30 Ph = 0.195; GA vs. GG: pooled OR = 1.17, 95% CI: 1.05–1.31, Ph = 0.123), especially in non-Caucasians (Table 2 and Fig 3). The association was stronger among females than males.

### Association between *NOS3* +894G>T polymorphism and migraine

**Fig 4:**
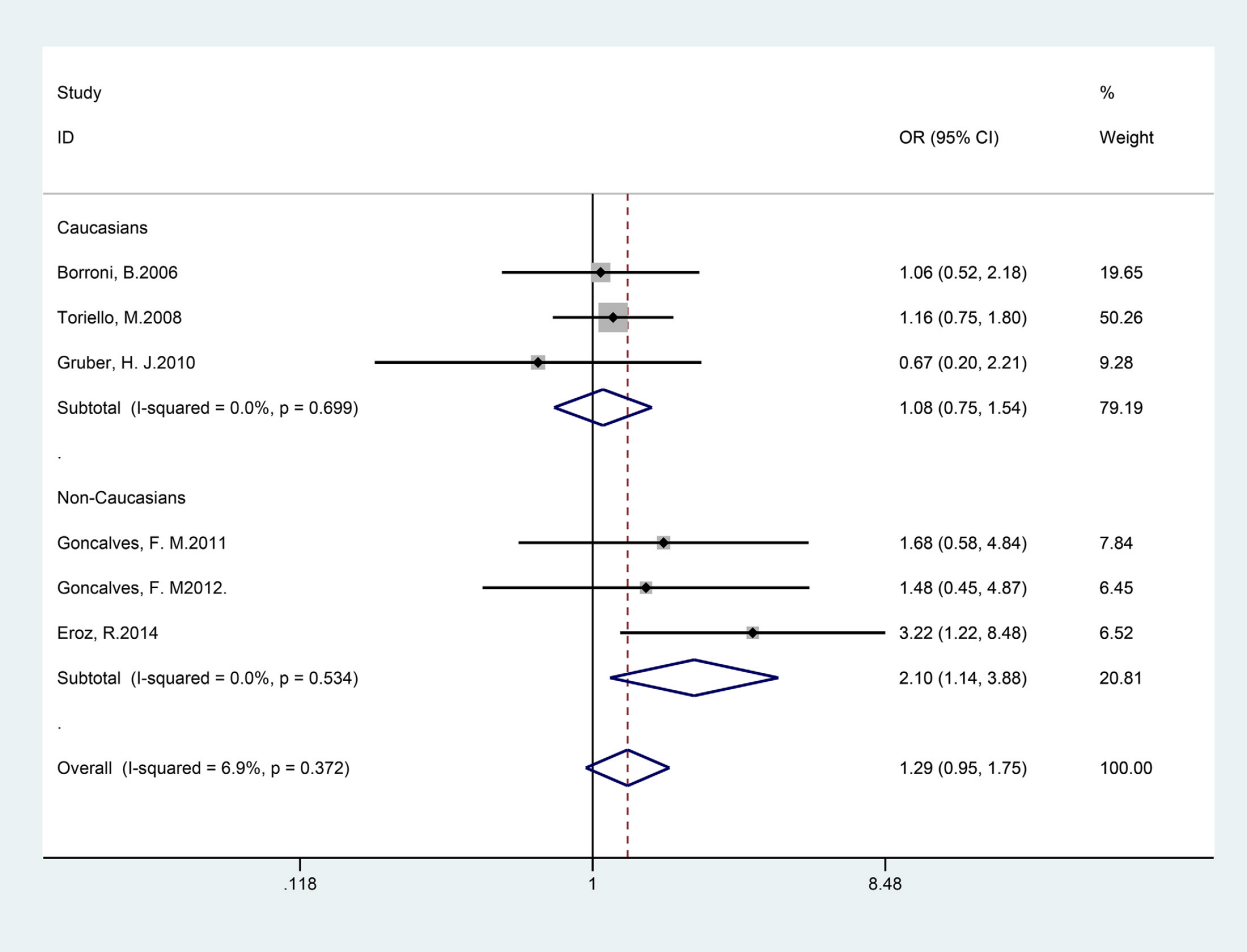
Forest plot of migraine risk associated with the *NOS3* +894G>T polymorphism stratified by ethnicity under the TT vs. GG model. The boxes and horizontal lines represent the OR and the corresponding 95% CI. The areas of the boxes indicate the weight (inverse of the variance). The diamonds correspond to the summary OR and 95% CI.

By pooling the six selected studies with 1055 genotyped migraine cases and 877 controls for *NOS3* +894G>T, no significant association between *NOS3* +894G>T polymorphism and migraine risk was observed under any of the genetic models examined. Subgroup analyses suggested that the T allele of the *NOS3* +894G>T variant increased the risk for migraine among non-Caucasians, which was driven by associations for MA (co-dominant model TT vs. GG: pooled OR = 2.10; 95% CI 1.14–3.88), as shown in Table 3 and Fig 4).

### Heterogeneity and sensitivity analyses

**Fig 5:**
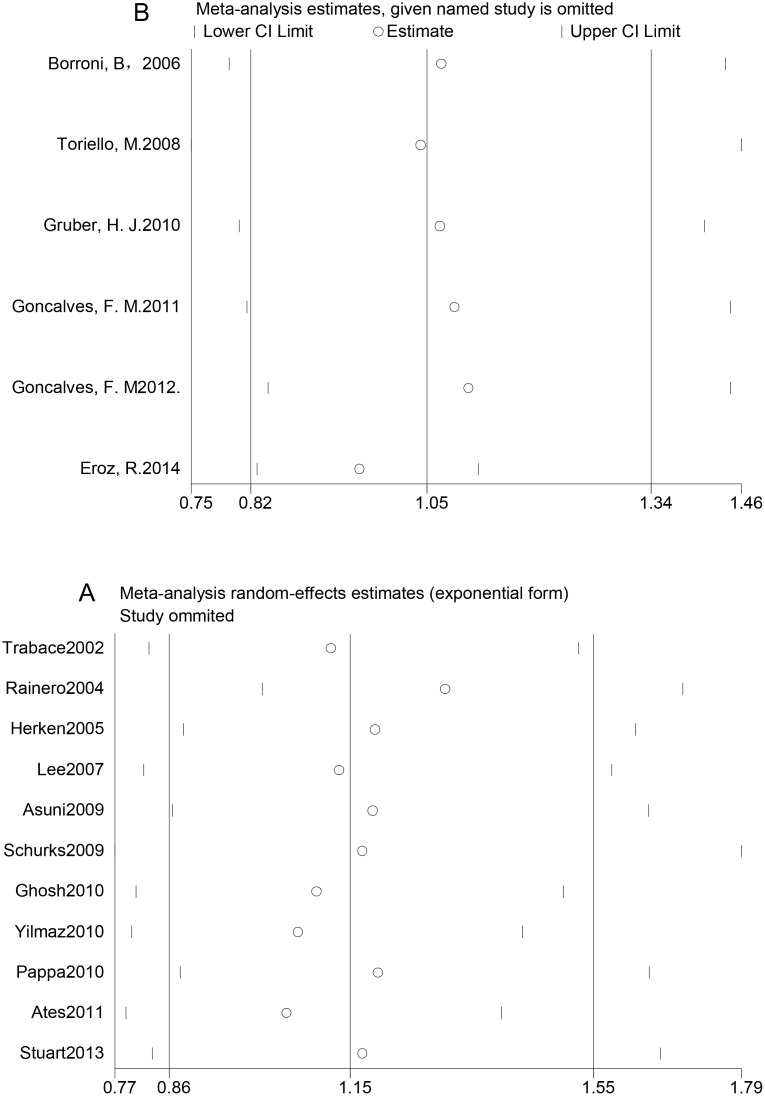
One-way sensitivity analyses of the associations between genetic polymorphisms and migraine risk. A: One-way sensitivity analysis of the association between the *TNF* –308G>A polymorphism and migraine risk under the GA vs. GG model. B: One-way sensitivity analysis of the association between the *NOS3* +894G>T polymorphism and migraine risk under the T vs. G model.

There was no significant heterogeneity among studies of *TNF* –308G>A polymorphism and MA (P > 0.10 for all genetic models). Nevertheless, the reverse effects were observed in studies of the *TNF* –308G>A polymorphism and migraine without aura (MO). Using Galbraith plots, we identified individual studies as important sources of heterogeneity [26, 28, 30, 31, 51], as shown in S1A and S1B Fig Among the five studies that were sources of heterogeneity, one had a low NOS scale, indicating low quality [51], and the other three had study populations with heterogeneous ethnic backgrounds [28, 30, 31]. Therefore, we performed sensitivity analyses by excluding studies that fell outside the margin set by the z score ± 2 standard deviation. We conducted sensitivity analysis to explore the potential sources of heterogeneity in the overall results. The initial ORs were not significantly influenced by sequential removal of each study from the total analysis (Fig 5A and 5B).

### Publication bias

**Fig 6:**
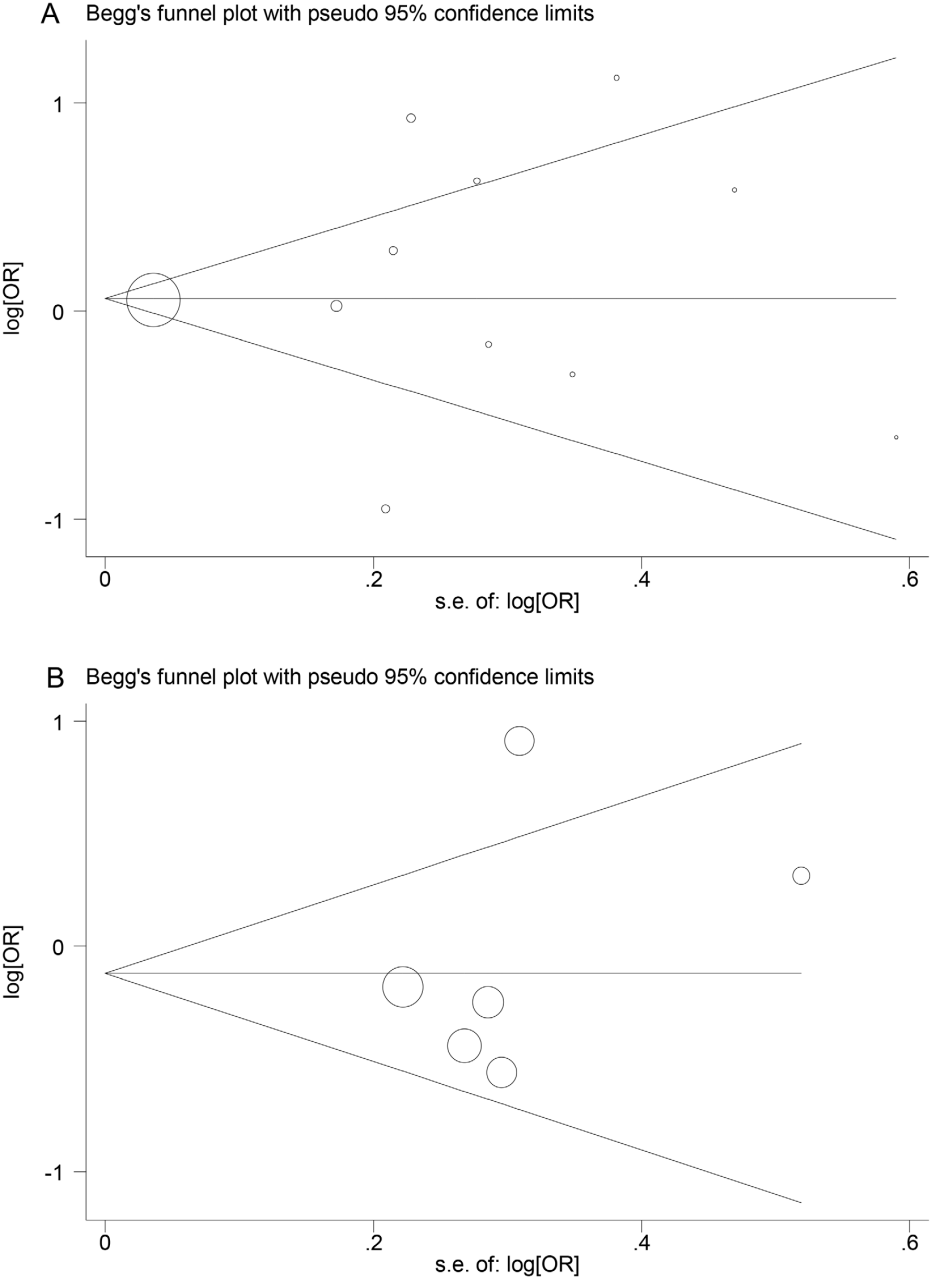
Begg’s funnel plots for genetic polymorphisms. A: Begg’s funnel plot for the *TNF* –308G>A polymorphism. B: Begg’s funnel plot for the *NOS3* +894G>T polymorphism.

Publication bias was determined using Begg’s funnel plots and Egger’s test. Neither Egger’s test nor Begg’s funnel plots indicated any significant publication bias (P = 0.699 and 1.000, respectively, for *TNF* –308G>A, P = 0.707 and 0.508, respectively, for *NOS3* +894G>T) (Fig 6A and 6B).

## Discussion

The overall results of this meta-analysis did not indicate associations between any of the polymorphisms investigated and migraine. However, the associations differed by ethnicity and clinical phenotype.

Among studies investigating the *TNF* –308G>A polymorphism, stratified analysis by ethnicity suggested an increased risk of migraine among non-Caucasians. When stratifying the data by type of migraine, we observed an increased risk of MA among both non-Caucasians and Caucasians, but especially among non-Caucasians.

Subgroup analyses suggested that the T allele of *NOS3* +894G>T variant increased the risk of migraine among non-Caucasians, which was driven by associations for MA. Assuming an allele T frequency of 10.64% among controls and using the TT vs. GT+GG genetic model, this study had about 84.3% power at a significance level of α = 0.05 to detect an effect size of 1.84 among non-Caucasians [57].

“Neurogenic inflammation” around the dural trigeminal afferents is known to play an important role in the generation of migraine attacks [7] and to contribute to the activation and sensitization of perivascular meningeal afferents during migraine attacks [58]. TNF-α and NO, potential noxious pain mediators in neurovascular inflammation, are involved in the initiation and maintenance of a migraine attack [59]. Furthermore, TNF-α contributes to the development of central sensitization by enhancing excitatory and reducing inhibitory currents and by activating induction of COX-2, which plays an important role in the development of inflammatory hyperalgesia [60], a phenomenon related to allodynia experienced by some migraineurs. In addition, Eroz et al. reported that patients with the *NOS3* +894G>T genotype GT have an increased risk of allodynia [51].

The levels of TNF-α and NO are under genetic control. An “A” at position –308 in the *TNF* promoter has been shown to be associated with a high level of TNF-α expression [61, 62]. A functional polymorphism hypothesis links TNF responses to certain *HLA* alleles, which have high linkage disequilibrium with *TNF*. Individuals carrying the “A” allele as part of the *HLA* Al, B8, DR3 haplotypes, which are population-specific within the human MHC region [63], may have higher levels of TNF-α expression. The different results obtained after stratifying by ethnicity may have been due to the influence of linkage disequilibrium pattern. Studies included in the present meta-analysis had similar results, with the exception of the study by Rainero et al. [26], in which subjects homozygous for the G allele had a higher risk of developing the disease. This discrepancy may have been due to differences in the ethnicity of the study populations.

The associations of the *TNF* –308G>A and *NOS3* +894G>T polymorphisms with predisposition to migraine have been examined in a number of studies, which reported inconsistent results. Due to the failure of independent association studies in identifying a role for the *TNF* –308G>A polymorphism in migraine, two meta-analyses were performed to reach definitive and reliable conclusions. Gu et al. [40] performed a meta-analysis associating the *TNF* –308G>A polymorphism with migraine based on six studies in Asian populations (985 cases and 958 controls); their results indicated that the *TNF* –308G>A polymorphism was associated with migraine risk in Asians. Subgroup analysis suggested that there was a statistically significant result for MA but not for MO. Another meta-analysis conducted by Schuerks et al. [64] based on 10 studies available for the *TNF* –308G>A polymorphism indicated no overall association with migraine. Subgroup analyses suggested that the “A” allele of the *TNF* –308G>A variant increases the risk of migraine among non-Caucasian populations, which was driven by associations for MO. In our study, subgroup analyses suggested that the risk of migraine with aura (MA) was increased among both Caucasians and non-Caucasians. Performance of a genome-wide association study (GWAS) enables identification of genetic variants in many multi-pathogenic diseases. To date, neither the *TNF* –308G>A nor the *NOS3 +894G>T* polymorphism has been included in GWAS.

Despite their plausible associations with migraine, this meta-analysis revealed no general associations between the *TNF* –308G>A or *NOS3* +894G>T polymorphism and migraine risk. There are two possible explanations for the observed overall lack of associations. Either, there may be really be no association and the positive results in some studies may have occurred by chance or due to certain study characteristics, or the pattern of association may be more complex and involve additional factors. In this context, considerations such as the heterogeneity among studies, different sources of controls, and variable sample sizes among the studies must be addressed.

Specifically, there were moderate to high degrees of heterogeneity among the studies investigating the association of the *TNF* –308G>A polymorphism with migraine and MO, which may have been due to differences between subgroups. Furthermore, assuming that the frequency of the “A” allele is 2.74% among controls and using the A vs. G genetic model, this study had only about 61.5% power at a significance level of = 0.05 to detect an effect size of 1.74 among non-Caucasians [57]. To achieve 80% power, the study would require 1522 cases. Moreover, with regard to *NOS3* +894G>T, not all information was available, which would have been valuable to draw clear associations with migraine among populations of different ethnicities.

Inherent bias, such as sampling bias, selection bias, publication bias, and within-study bias, cannot be avoided in a meta-analysis of observational studies. The following biases must be taken into consideration when interpreting the results of the present study. First, all of the studies included in the meta-analysis were published in English, so selection bias may have occurred. Second, we did not include fugitive literature, including academic dissertations and conference papers, so there may have been bias in provision of data. Third, there was a lack of unified sources of controls. The controls were selected from three sources: hospital-based, healthy population, and family members of the migraineurs, so selection bias may have occurred especially among the studies with hospital-based controls. Fourth, there was heterogeneity across the studies, which may have masked significance in the present study. Another limitation of our study was that we did not analyze the gene-to-gene or gene-environment interactions that may contribute to modification of migraine risk due to a lack of data among the existing publications eligible for this meta-analysis.

## Conclusions

In summary, the general results of our meta-analysis do not support a strong overall effect, but provide statistical evidence that the *TNF* –308G>A polymorphism in the *TNF* region may represent a risk for migraine among non-Caucasians and MA among both Caucasians and non-Caucasians. The *NOS3* +894G>T polymorphism of eNOS may represent a risk for migraine among non-Caucasians. However, given the heterogeneity among study designs, the results of this meta-analysis should be interpreted with caution. Further cohort studies based on pedigree are warranted to evaluate population-specific effects, including population stratification.

## Supporting Information

S1 Checklist Meta-analysis on Genetic Association Studies Checklist | PLoS ONE. (DOCX)

S2 Checklist PRISMA. 2009. Checklist. (DOC)

S1 Dataset List of the excluded studies of *TNF* –308G>A. (XLSX)

S1 Fig Galbraith plots for the genetic polymorphisms. A: S1A Fig Galbraith plot for *TNF* –308G>A. B: S1B Fig Galbraith plot for *NOS3* +894G>T.
(EPS)

S1 Table Allele and genotype frequencies according to *TNF* –308G>A. MA: migraine with aura; MO: migraine without aura
(DOCX)

S2 Table Allele and genotype frequencies according to *NOS3* +894G>T. MA: migraine with aura; MO: migraine without aura
(DOCX)
