# Supplementary material for: Tumor Necrosis Factor (TNF) –308G>A, Nitric Oxide Synthase 3 (NOS3) +894G>T Polymorphisms and Migraine Risk: A Meta-Analysis
Source: PLoS One. 2015 Jun 22;10(6):e0129372. doi: 10.1371/journal.pone.0129372 (PMC4476787; doi:10.1371/journal.pone.0129372)
Supplement: S1 Table — MA: migraine with aura; MO: migraine without aura (DOCX) [file pone.0129372.s005.docx]

**Supporting Information Table S1. Allele and genotype frequencies according to *TNF* –308G>A.**

| Author(ref.) | Participants | Disease | Study size | Allele frequencies, n(%) | | Genotype frequencies, n(%) | | |
| --- | --- | --- | --- | --- | --- | --- | --- | --- |
|  |  |  |  | G | A | GG | GA | AA |
| Trabace 2002[42] | all | controls | 101 | 189(93.6) | 13(6.4) | 90(89.1) | 9(8.9) | 2(2.0) |
|  |  | Migraine | 79 | 146(92.4) | 12(7.6) | 67(84.8) | 12(15.2) | 0(0.0) |
|  |  | MA | 32 | 57(89.1) | 7(10.9) | 25(78.1) | 7(21.9) | 0(0.0) |
|  |  | MO | 47 | 89(94.7) | 5(5.3) | 42(89.4) | 5(10.6) | 0(0.0) |
|  | females | controls | 45 | 85(94.4) | 5(5.6) | 41(91.1) | 3(6.7) | 1(2.2) |
|  |  | migraine | 62 | 114(91.9) | 10(8.1) | 52(83.9) | 10(16.1) | 0(0.0) |
|  |  | MA | 27 | 48(88.9) | 6(11.1) | 21(77.8) | 6(22.2) | 0(0.0) |
|  |  | MO | 35 | 66(94.3) | 4(5.7) | 31(88.6) | 4(11.4) | 0(0.0) |
|  | males | controls | 56 | 104(92.9) | 8(7.1) | 49(87.5) | 6(10.7) | 1(1.8) |
|  |  | migraine | 17 | 32(94.1) | 2(5.9) | 15(88.2) | 2(11.8) | 0(0.0) |
|  |  | MA | 5 | 9(90.9) | 1(10.0) | 4(80.0) | 1(20.0) | 0(0.0) |
|  |  | MO | 12 | 23(95.8) | 1(4.2) | 11(91.7) | 1(8.3) | 0(0.0) |
| Rainero 2004[17] | all | controls | 306 | 502(82.0) | 110(18.0) | 207(67.6) | 88(28.8) | 11(3.6) |
|  |  | Migraine | 299 | 554(92.6) | 44(7.4) | 256(85.6) | 42(14.0) | 1(0.3) |
|  |  | MA | 38 | 66(86.8) | 10(13.2) | 28(73.7) | 10(26.3) | 0(0.0) |
|  |  | MO | 261 | 488(93.5) | 34(6.5) | 228(87.4) | 32(12.3) | 1(0.4) |
|  | females | controls | 231 | 382(82.7) | 80(17.3) | 158(68.4) | 66(28.6) | 7(3.0) |
|  |  | migraine | 215 | 401(93.3) | 29(6.7) | 186(86.5) | 29(13.5) | 0(0.0) |
|  | males | controls | 75 | 120(80.0) | 30(20.0) | 49(65.3) | 22(29.3) | 4(5.3) |
|  |  | migraine | 84 | 153(91.1) | 15(8.9) | 70(83.3) | 13(15.5) | 1(1.2) |
| Herken 2005[33] | all | controls | 62 | 115(92.7) | 9(7.3) | 53(85.5) | 9(14.5) | 0(0.0) |
|  |  | Migraine | 60 | 113(94.2) | 7(5.8) | 54(90.0) | 5(8.3) | 1(1.7) |
|  |  | MA | 40 | 76(95.0) | 4(5.0) | 36(90.0) | 4(10.0) | 0(0.0) |
|  |  | MO | 20 | 37(92.5) | 3(7.5) | 18(90.0) | 1(5.0) | 1(5.0) |
| Lee 2007[24] | all/females | controls | 382 | 717（93.8） | 47（6.2） | 338（88.5） | 41(10.7) | 3(0.8) |
|  |  | migraine | 439 | 815（92.8） | 63（7.2） | 377（85.9） | 61(13.9) | 1(0.2) |
|  |  | MA | 65 | 119（91.5） | 11（8.5） | 54（83.1） | 11(16.9) | 0(0.0) |
|  |  | MO | 327 | 608（93.0） | 46（7.0） | 282（86.2） | 44(13.5) | 1(0.3) |
| Asuni 2009[27] | all | controls | 278 | 526（94.6） | 30（5.4） | 249（89.6） | 28(10.1) | 1(0.4) |
|  |  | MO | 299 | 570（95.3） | 28（4.7） | 272（91.0） | 26(8.7) | 1(0.3) |
|  | females | controls | 144 | 273（94.8） | 15（5.2） | 129（89.6） | 15(10.4) | 0(0.0) |
|  |  | MO | 261 | 497（95.2） | 25（4.8） | 237（90.8） | 23(8.8) | 1(0.4) |
|  | males | controls | 134 | 253（94.4） | 15（5.6） | 120（89.6） | 13(9.7) | 1(0.7) |
|  |  | MO | 38 | 73（96.1） | 3（3.9） | 35（92.1） | 3(7.9) | 0(0.0) |
| Schurks 2009[37] | all/females | controls | 20，425 | 33,771（82.7） | 7079（17.3） | 13,947（68.3） | 5877(28.8) | 601(2.9) |
|  |  | migraine | 4577 | 7531（82.3） | 1623（17.7） | 3081（67.3） | 1369(29.9) | 127(2.8) |
|  |  | MA | 1275 | 2060（80.8） | 490（19.2） | 827（64.9） | 406(31.8) | 42(3.3) |
|  |  | MO | 1951 | 3240（83.0） | 662（17.0） | 1346（69.0） | 548(28.1) | 57(2.9) |
| Ghosh 2010[22] | all | controls | 216 | 406（94.0） | 26（6.0） | 191（88.4） | 24(11.1) | 1(0.5) |
|  |  | Migraine | 216 | 391（90.5） | 41（9.5） | 175（81.0） | 41(19.0) | 0(0.0) |
|  |  | MA | 84 | 149（88.7） | 19（11.3） | 65（77.4） | 19(22.6) | 0(0.0) |
|  |  | MO | 132 | 242（91.7） | 22（8.3） | 110（83.3） | 22(16.7) | 0(0.0) |
|  | females | controls | 152 | 285（93.8） | 19（6.3） | 134（88.2） | 17(11.2) | 1(0.7) |
|  |  | migraine | 152 | 273（89.8） | 31（10.2） | 121（79.6） | 31(20.4) | 0(0.0) |
|  |  | MA | 63 | 109（86.5） | 17（13.5） | 46（73.0） | 17(27.0) | 0(0.0) |
|  |  | MO | 89 | 164（92.1） | 14（7.9） | 75（84.3） | 14(15.7) | 0(0.0) |
|  | males | controls | 64 | 121（94.5） | 7（5.5） | 57（89.1） | 7(10.9) | 0(0.0) |
|  |  | migraine | 64 | 118（92.2） | 10（7.8） | 54（84.4） | 10(15.6) | 0(0.0) |
|  |  | MA | 21 | 40（95.2） | 2（4.8） | 19（90.5） | 2(9.5) | 0(0.0) |
|  |  | MO | 43 | 78（90.7） | 8（9.3） | 35（81.4） | 8(18.6) | 0(0.0) |
| Yilmaz 2010[19] | all | controls | 96 | 174（90.6） | 18（9.4） | 79（82.3） | 16(16.7) | 1(1.0) |
|  |  | MO | 67 | 97（72.4） | 37（27.6） | 37（55.2） | 23(34.3) | 7(10.4) |
|  | females | controls | 83 | 152（91.6） | 14（8.4） | 70（84.3） | 12(14.5) | 1(1.2) |
|  |  | MO | 57 | 83（72.8） | 31（27.2） | 32（56.1） | 19(33.3) | 6(10.5) |
|  | males | controls | 13 | 22（84.6） | 4（15.4） | 9（69.2） | 4(30.8) | 0(0.0) |
|  |  | MO | 10 | 14（70.0） | 6（30.0） | 5（50.0） | 4(40.0) | 1(10.0) |
| Pappa 2010[26] | all | controls | 178 | 321（90.2） | 35（9.8） | 145（81.5） | 31(17.4) | 2(1.1) |
|  |  | MO | 103 | 192（93.2） | 14（6.8） | 89（86.4） | 14(13.6) | 0(0.0) |
|  | females | controls | 60 | 107（89.2） | 13（10.8） | 48（80.0） | 11(18.3) | 1(1.7) |
|  |  | MO | 57 | 105（92.1） | 9（7.9） | 48（84.2） | 9(15.8) | 0(0.0) |
|  | males | controls | 118 | 214（90.7） | 22（9.3） | 97（82.2） | 20(16.9) | 1(0.8) |
|  |  | MO | 46 | 87（94.6） | 5（5.4） | 41（89.1） | 5(10.9) | 0(0.0) |
| Ates 2011[32] | all | controls | 202 | 364（90.1） | 40（19.8） | 162（80.2） | 40（19.8） | 0（0.0） |
|  |  | migriane | 203 | 328（80.8） | 78（19.2） | 125（61.6） | 78（38.4） | 0（0.0） |
| Stuart 2013[25] | all | controls | 345 | 557（80.7） | 133（19.3） | 230（66.7） | 97（28.1） | 18（5.2） |
|  |  | migraine | 340 | 535（79.9） | 135（20.1） | 220（65.7） | 95（28.4） | 20（6.0） |
|  |  | MA | 229 | 359（79.8） | 91（20.2） | 149（66.2） | 61（27.1） | 15（6.7） |
|  |  | MO | 111 | 175（80.3） | 43（19.7） | 71（65.1） | 33（30.3） | 5（4.6） |
|  | females | controls | 269 | 434（81.9） | 96（18.1） | 182（68.7） | 70（26.4） | 13（4.9） |
|  |  | migraine | 249 | 397（79.7） | 101（20.3） | 163（65.5） | 71（28.5） | 15（6.0） |
|  | males | controls | 76 | 121（79.6） | 31（20.4） | 50（65.8） | 21（27.6） | 5（6.6） |
|  |  | migraine | 91 | 140（77.8） | 40（22.2） | 55（61.1） | 30（33.3） | 5（5.6） |

MA: migraine with aura; MO: migraine without aura
