# Supplementary material for: Tumor Necrosis Factor (TNF) –308G>A, Nitric Oxide Synthase 3 (NOS3) +894G>T Polymorphisms and Migraine Risk: A Meta-Analysis
Source: PLoS One. 2015 Jun 22;10(6):e0129372. doi: 10.1371/journal.pone.0129372 (PMC4476787; doi:10.1371/journal.pone.0129372)
Supplement: S2 Table — MA: migraine with aura; MO: migraine without aura (DOCX) [file pone.0129372.s006.docx]

**Supporting Information Table S2. Allele and genotype frequencies according to *NOS3* +894G>T.**

| Author(ref.) | Participants | Disease | Study size | Allele frequencies, n(%) | | Genotype frequencies, n(%) | | |
| --- | --- | --- | --- | --- | --- | --- | --- | --- |
|  |  |  |  | G | T | GG | GT | TT |
| Borroni, B，2006[13] | all | controls | 125 | 168 (67.2) | 82(32.8) | 59(47.2) | 50(40.0) | 16(12.8) |
|  |  | Migraine | 156 | 213(68.3) | 99(31.7) | 80(51.3) | 53(34.0) | 23(14.7) |
|  |  | MA | 53 | 64(60.4) | 42(39.6) | 24(45.3) | 16(30.2) | 13(24.5) |
|  |  | MO | 103 | 149(72.3) | 57(27.7) | 56(54.4) | 37(35.9) | 10(9.7) |
| Toriello, M.2008[14] | all | controls | 341 | 385(56.1) | 297(43.9) | 104(30.5) | 177(51.9) | 60(17.6) |
|  |  | Migraine | 337 | 371(55) | 303(45) | 103(30.5) | 165(49.0) | 69(20.5) |
|  |  | MA | 188 | 200(52.9) | 176(47.1) | 53(28.2) | 94(50.0) | 41(21.8) |
|  |  | MO | 149 | 171(57.4) | 127(42.6) | 50(33.5) | 71(47.7) | 28(18.8) |
| Gruber, H. J.2010[15] | all | controls | 76 | 96(63.2) | 56(36.8) | 31(40.8) | 34(44.7) | 11(14.5) |
|  |  | Migraine | 54 | 70(64.8) | 38(35.2) | 21(38.9) | 28(51.9) | 5(9.2) |
|  |  | MA | 20 | 42(61.8) | 26(38.2) | 13(38.3) | 16(47) | 5(14.7) |
|  |  | MO | 34 | 28(70.0) | 12(30.0) | 8(40.0) | 12(60.0) | 0(0) |
| Goncalves, F. M.2011[16] | all/females | controls | 117 | 173（74.0） | 61（26.0） | 61（52.0） | 51(44.0) | 5(40) |
|  |  | migraine | 178 | 272（76.0） | 84（24.0） | 109（61.0） | 54(30.0) | 15(9.0) |
|  |  | MA | 44 | 69（78.0） | 19（22.0） | 29（66.0） | 11(25.0) | 4(9.0) |
|  |  | MO | 134 | 203（76.0） | 65（24.0） | 80（60.0） | 43(32.0) | 11(8.0) |
| Goncalves, F. M.2012[17] | all | controls | 99 | 146（74.0） | 52（26.0） | 51（52.0） | 44(44.0) | 4(4.0) |
|  |  | migriane | 150 | 234（78.0） | 66（22.0） | 95（64.0） | 44(29.0) | 11(7.0) |
|  |  | MA | 43 | 67(78.0) | 19(22.0) | 28(65.0) | 11(26.0) | 4(9.0) |
|  |  | MO | 107 | 167(78.0) | 47(22.0) | 67(63.0) | 33(31.0) | 7(5.0) |
| Eroz, R.2014[18] | all | controls | 123 | 178（72.4） | 68（27.6） | 62（50.4） | 54(43.9) | 7(5.7) |
|  |  | Migraine | 176 | 204(58.0) | 148（42.0） | 44（25.0） | 116(65.9) | 16(9.1) |
|  |  | MA | 92 | 104（56.5） | 80（43.5） | 20（21.7） | 64(69.6) | 8(8.7) |
|  |  | MO | 84 | 100（59.5） | 68（40.5） | 24（28.6） | 52(61.9) | 8(9.5) |

MA: migriane with aura; MO: migraine without aura
